# Supplementary material for: Thin Film Biocomposite Membrane for Forward Osmosis Supported by Eggshell Membrane
Source: Membranes (Basel). 2022 Jan 30;12(2):166. doi: 10.3390/membranes12020166 (PMC8879599; doi:10.3390/membranes12020166)
Supplement: Supplementary file 1 [file membranes-12-00166-s001.zip › membranes-1556799-supplementary.pdf]

membranes

Supplementary Information

Thin film Biocomposite Membrane for Forward Osmosis Supported by Eggshell Membrane

Teayeop Kim[1,†], Sunho Park[2,3,†], Yoonkyung Lee[1], Jangho Kim[2,3,*] and Kyunghoon Kim[1,*]

[1] School of Mechanical Engineering, Sungkyunkwan University, Suwon 16419, Republic of Korea; skkty@skku.edu (T. K.), yi9257kr@skku.edu (Y.L.)
[2] Department of Rural and Biosystems Engineering, Chonnam National University, Gwangju 61186, Republic of Korea; preference9330@gmail.com (S. P.)
[3] Interdisciplinary Program in IT-Bio Convergence System, Chonnam National University, Gwangju, 61186, Republic of Korea
* Correspondence: kenkim@skku.edu (K. K.), rain2000@jnu.ac.kr (J. K.).
† These authors equally contribute to this paper.

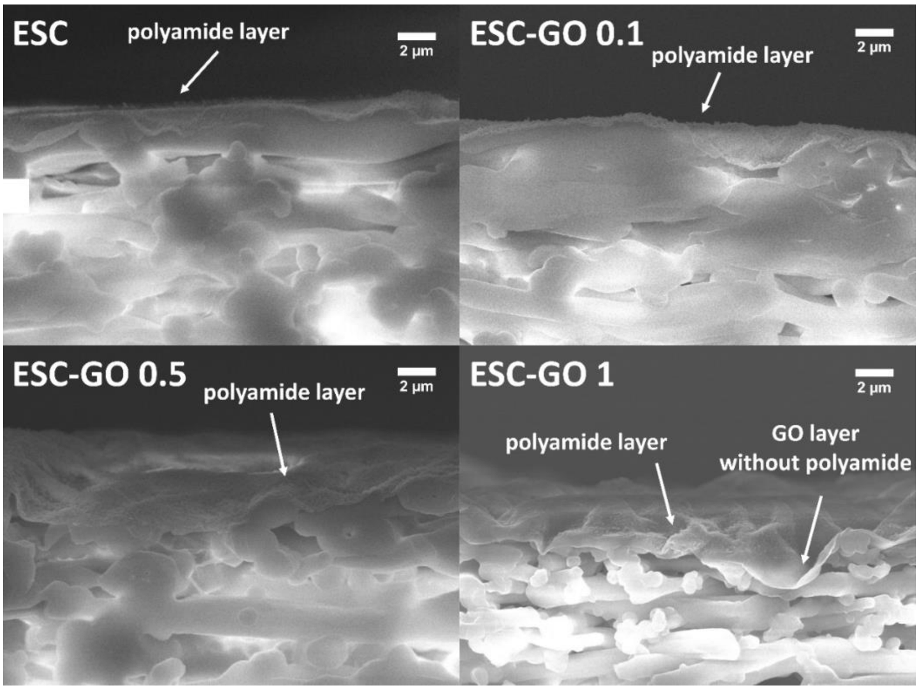

Figure S1. FESEM image of ESC and ESC-GO membranes at polyamide layer.

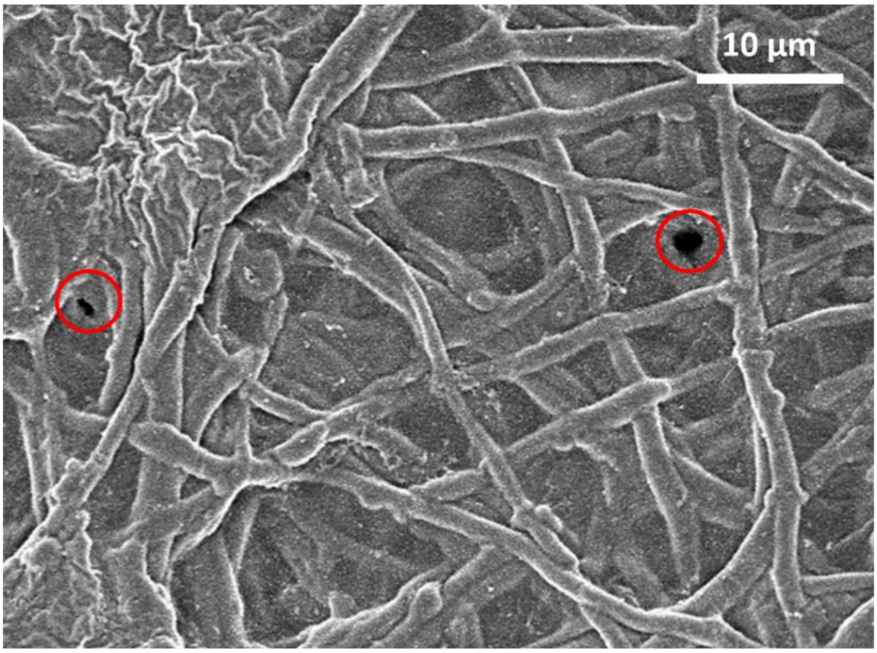

Figure S2. Defects at polyamide layer in ESC membranes.

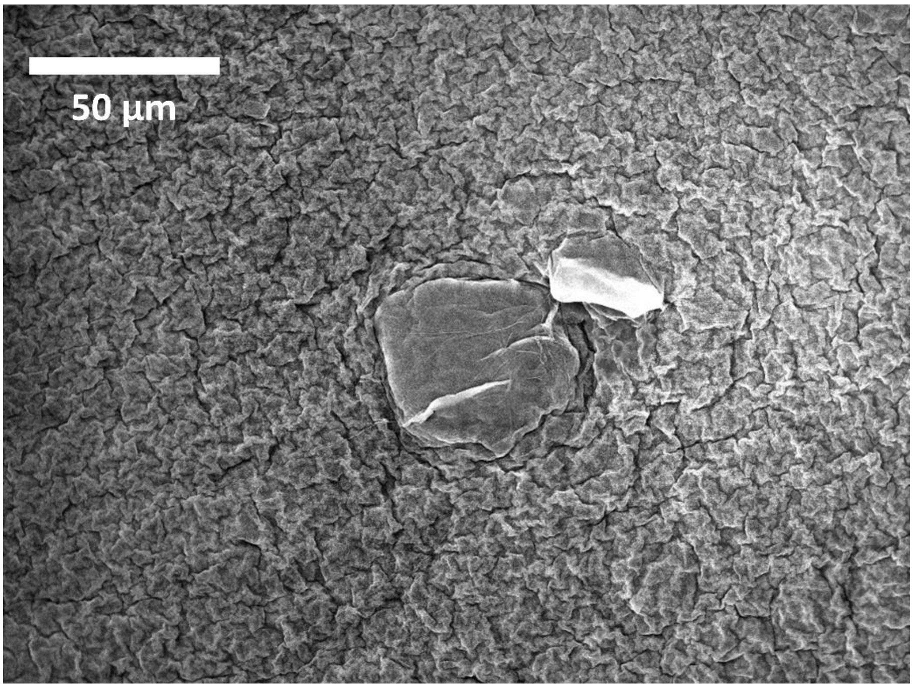

Figure S3. Large agglomeration of GO in ESM-GO 1.
